# Dynamic bedside lung ultrasound in the diagnosis and management of neonatal lung abscess: a case report

**DOI:** 10.3389/fped.2026.1785149

**Published:** 2026-05-20

**Authors:** Li-Juan Peng, Shu-Yue Tao, Lin Li

**Affiliations:** 1 Department of Ultrasound, Suining Central Hospital, Sichuan, China; 2 Department of Pediatrics, Suining Central Hospital, Sichuan, China

**Keywords:** lung abscess, lung ultrasound, Neonatal pneumonia, Puncture treatment, Ultrasound-guided

## Abstract

Neonatal pneumonia (NP) is a common disease, but neonatal lung abscess is a relatively rare but serious complication of neonatal pneumonia. At present, lung ultrasound (LUS) is widely recommended for the diagnosis of pneumonia in neonates and children, but there are few reports of neonatal lung abscess (NLA) ultrasound. In this paper, ultrasound was used to completely record a 26-day old neonate who was hospitalized due to Staphylococcus aureus infection pneumonia. During the treatment process, the pneumonia developed into lung abscess, and puncture and drainage were performed under ultrasound monitoring. After ultrasound intervention and active medical symptomatic treatment, the clinical symptoms and lung ultrasound images were significantly improved, which greatly reduced the x-ray intake during the monitoring of lung disease. After 29 days of treatment, the neonate was discharged. Ultrasound for the dynamic observation of neonatal lung disease bedside, so that clinicians can pay close attention to the treatment effect, and choose the appropriate treatment, to provide reference information for the monitoring and treatment of neonatal lung abscess.

## Introduction

Neonatal lung abscess (NLA) is an uncommon but potentially life-threatening complication of severe pulmonary infection in newborns, characterized by localized parenchymal necrosis and cavitary formation containing purulent material. While neonatal pneumonia (NP) is a common contributor to respiratory morbidity in both term and preterm infants, progression to lung abscess during the neonatal period is rare and infrequently reported, leading to limited clinical recognition and delayed diagnosis. Documented risk factors for NLA include prematurity, immature immune function, aspiration events, and severe or inadequately controlled pulmonary infections. The pathogens most frequently implicated comprise Staphylococcus aureus, Escherichia coli, Klebsiella pneumoniae, and other Gram-negative organisms (1, 2).

Historically, chest radiography and computed tomography (CT) have been the primary imaging modalities for diagnosis, enabling visualization of consolidation, cavitation, and air–fluid levels. However, their use in neonates is constrained by exposure to ionizing radiation, limited sensitivity for early lesions, and logistical challenges associated with transporting critically ill or mechanically ventilated infants. Although CT provides high-resolution anatomical detail and remains a reference standard for complex pulmonary infections, its routine or emergent use in neonatal populations is generally discouraged (3–5).

In recent years, lung ultrasound (LUS) and point-of-care ultrasound (POCUS) have gained acceptance as bedside, radiation-free imaging modalities for NP evaluation. Prospective and multicenter studies indicate that LUS exhibits high sensitivity and specificity in diagnosing neonatal pneumonia, detecting pulmonary consolidation, dynamic air bronchograms, pleural abnormalities, and inflammatory changes often before they are apparent on conventional radiographs (6–8). Consequently, LUS has been integrated into clinical practice guidelines and expert consensus statements for diagnosis and monitoring of neonatal and pediatric pneumonia (9). Beyond common neonatal pulmonary conditions such as respiratory distress syndrome, pneumonia, and atelectasis, emerging evidence supports the role of ultrasound in assessing complicated pulmonary infections, including NLA. Characteristic sonographic findings of lung abscess include heterogeneous consolidation, hypoechoic or anechoic cavitary lesions lacking internal vascularity, and dynamic evolution during treatment. Importantly, POCUS facilitates early detection, longitudinal monitoring, and ultrasound-guided interventions such as percutaneous aspiration or drainage, reducing reliance on CT imaging and minimizing radiation exposure (1, 10, 11).

In pediatric emergency and critical care settings, POCUS allows rapid bedside identification of lung abscess, supporting timely escalation of antimicrobial therapy or interventional management in infants with acute respiratory deterioration (10). Advances in ultrasound-guided diagnostic and therapeutic strategies further underscore the expanding role of LUS in evaluating complex neonatal lung pathology and guiding individualized clinical decision-making (11). Despite widespread adoption of LUS for NP, systematic documentation of sonographic findings throughout the evolution from pneumonia to abscess formation and subsequent resolution remains limited. In this report, we describe a preterm infant in whom serial lung ultrasound examinations captured disease progression, abscess formation, interventional management, and ultimate resolution, highlighting the comprehensive diagnostic and clinical utility of LUS in managing NLA.

## Case presentation

A male preterm infant was delivered vaginally at 35^+2^ weeks of gestation, with a birth weight of 2.3 kg. Apgar scores were 9, 10, and 10 at 1, 5, and 10 min, respectively. Serial prenatal ultrasonographic evaluations revealed no evidence of fetal pulmonary abnormalities, and the amniotic fluid, umbilical cord, and placenta were unremarkable on antenatal imaging. The maternal history was notable for adequately treated syphilis. On postnatal day 26 (weight 2.4 kg), the infant was admitted 6 hours after an episode of milk aspiration. One week prior, after exposure to cold, the infant developed reduced oral intake and intermittent cough without nasal obstruction or rhinorrhea. Six hours before admission, choking during feeding was followed by pallor and lethargy, without vomiting or abdominal distension.

The infant presented with lip cyanosis, mild tachypnea, and fluctuating oxygen saturation around 80% upon admission. Immediate airway clearance was performed, revealing scant blood-tinged secretions. Endotracheal intubation was then performed, and mechanical ventilation was initiated in assist-control (A/C) mode with the following settings: peak inspiratory pressure (PIP) 20 cmH_2_O, positive end-expiratory pressure (PEEP) 5 cmH_2_O, respiratory rate 35 breaths/min, fraction of inspired oxygen (FiO_2_) 60%, and inspiratory time (Ti) 0.5 s. Following intubation, the patient's oxygen saturation increased to 90%, with improved skin color.

**Figure 1:**
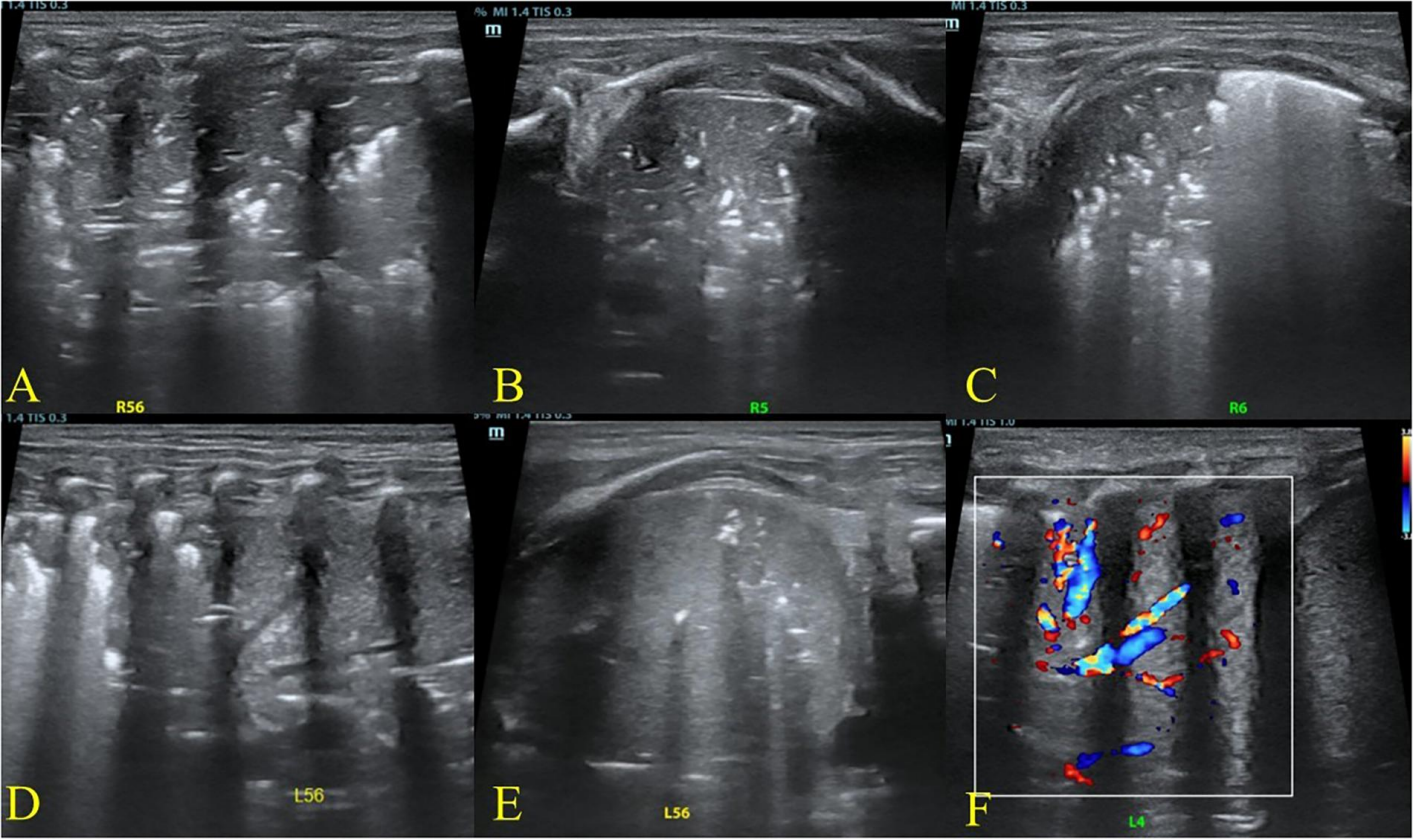
Baseline lung ultrasound findings at admission demonstrating bilateral posterior–lateral consolidation with intralesional vascularity. Initial ultrasound at admission demonstrated extensive consolidation in the lateral and posterior regions of both lungs. Air bronchograms were more prominent on the right than on the left. Color Doppler revealed vascular signals within the consolidated areas, consistent with pneumonia. **(A–C)** Longitudinal and transverse views of the right lung, zones 5–6; (**D–E)** longitudinal and transverse views of the left lung, zones 5–6; **(F)** color Doppler showing vascularity within the consolidated region.

**Figure 2:**
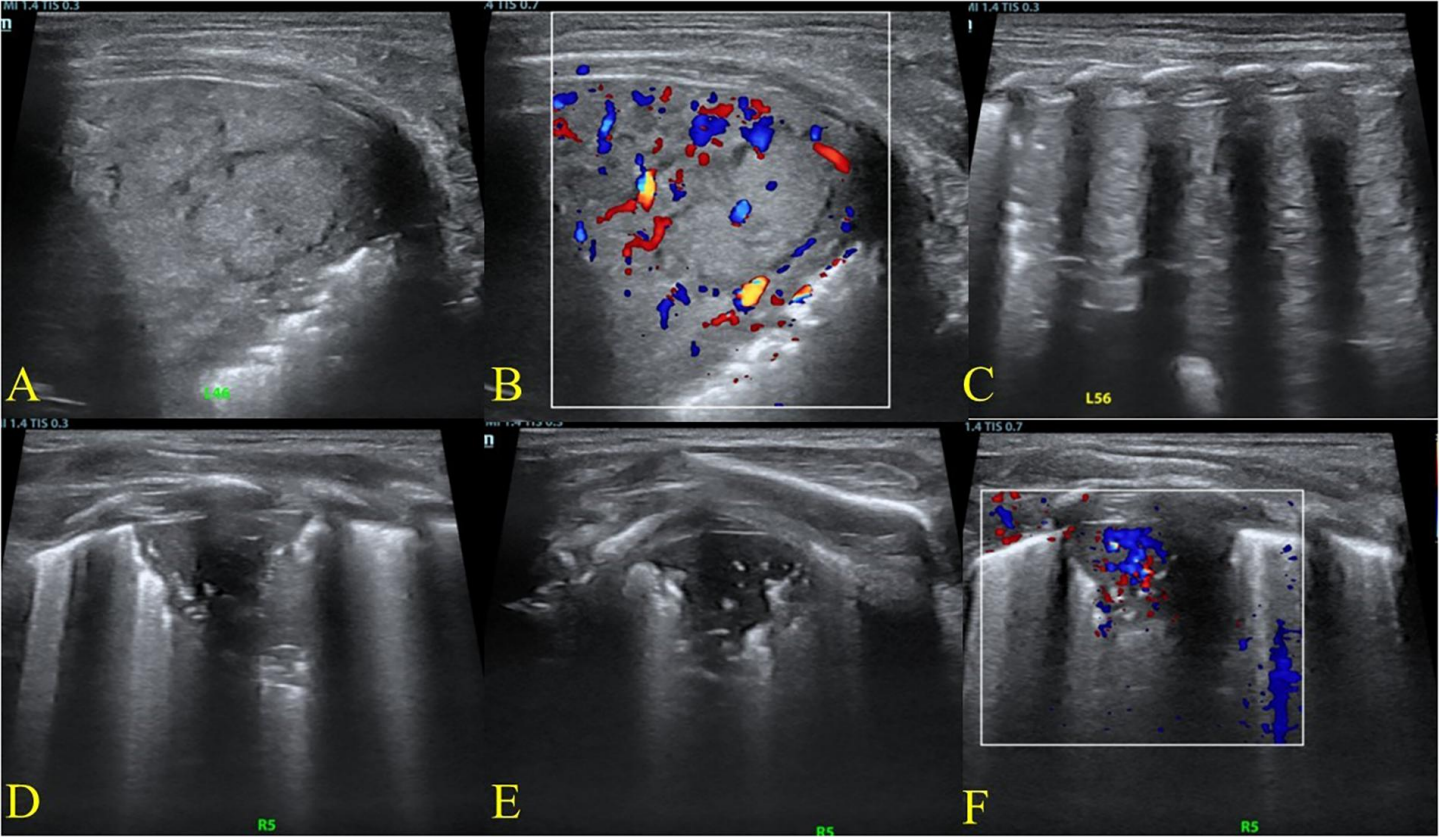
Serial lung ultrasound findings on hospital day 2 under conventional mechanical ventilation. Ultrasound on hospital day 2, under conventional mechanical ventilation, showed persistent heterogeneous consolidation of the left lung with detectable intralesional blood flow **(A–C)**. The right lung consolidation had markedly decreased, limited to the posterior upper region, with visible air bronchograms and blood flow signals **(D–F)**.

Initial LUS (Figure 1) revealed extensive bilateral consolidation in the lateral and posterior regions (bilateral range exceeds 50% of lung lobes), with more pronounced air bronchograms on the right. Doppler demonstrated blood flow in both areas, consistent with pneumonia. Laboratory findings showed leukopenia (WBC 2.8 × 10^9^/L), thrombocytopenia (PLT 30 × 10^9^/L), and anemia (Hb 94 g/L), with elevated CRP and PCT (CRP 12.02 mg/L, PCT 0.81 ng/mL). Antimicrobial therapy was initiated with flucloxacillin (60 mg, IV drip, Tid) in combination with cefoperazone–sulbactam sodium (120 mg, IV drip, Tid). On hospital day 2, LUS (Figure 2) showed persistent left lung consolidation (with no reduction in range) with heterogeneous echotexture and preserved vascularity, while right lung consolidation was reduced to the posterior upper region. Laboratory findings demonstrated a persistent elevation in CRP levels and a decline in platelet count. Ultrasonography revealed heterogeneous parenchymal changes in the left lung. Sputum Targeted next-generation sequencing (tNGS) identified Staphylococcus aureus. Accordingly, the antimicrobial regimen was adjusted to vancomycin (36 mg, IV drip, Tid) combined with cefoperazone–sulbactam sodium (120 mg, IV drip, Tid).

**Figure 3:**
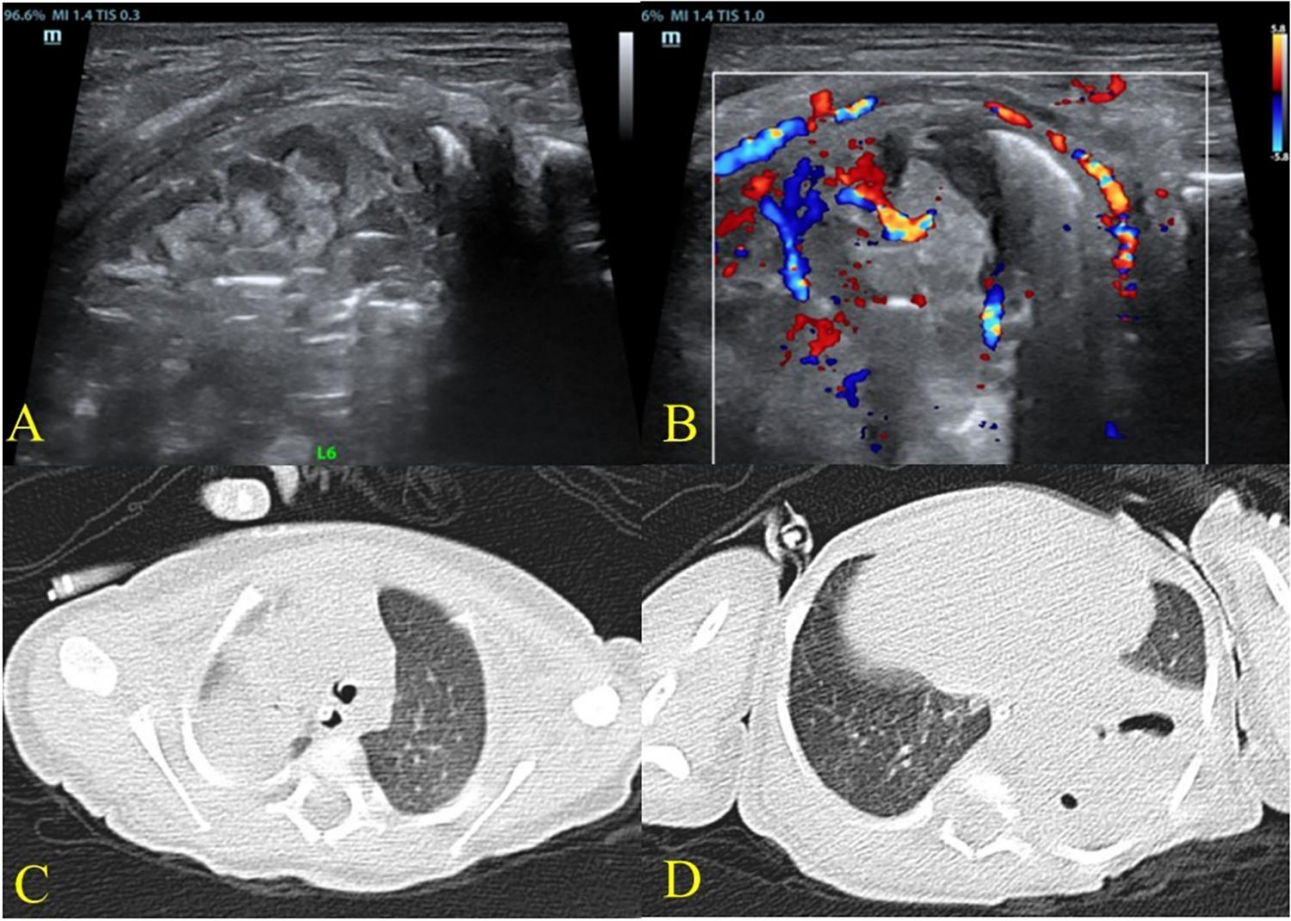
Lung ultrasound and chest CT findings on hospital day 4 demonstrating persistent left lung consolidation with suspected abscess formation. On hospital day 4, under conventional mechanical ventilation, ultrasound revealed persistent left lung consolidation with heterogeneous echotexture **(A)** Multiple strip-like anechoic areas without blood flow were observed within the consolidation, while vascular signals remained detectable in surrounding consolidated tissue **(B)**, suggestive of pneumonia with partial abscess formation. Chest CT demonstrated multiple areas of ground-glass opacity and consolidation in both lungs, more prominent in the right upper lobe **(C)** and left lower lobe **(D)** Air–fluid levels were visible within the left lower lobe consolidation, consistent with inflammation and suspected pulmonary abscess formation.

On hospital day 4, LUS (Figures 3A,B) revealed heterogeneous left lung consolidation (with no reduction in range) with multiple avascular hypoechoic areas, suggesting abscess formation, while right lung consolidation was the same as the day 2; On the same day, CT (Figures 3C,D) confirmed consolidation with air–fluid levels in the left lower lobe, suggesting pneumonia with lung abscess. On hospital day 6, fiberoptic bronchoscopy with bronchoalveolar lavage (BAL) was performed. Analysis of bronchoalveolar lavage fluid using tNGS detected Staphylococcus aureus and Mycoplasma pneumoniae. Consequently, azithromycin oral suspension (24 mg, QD) was added to the treatment regimen and administered for five consecutive days. On hospital day 10, the patient's clinical condition had stabilized. Mechanical ventilation was discontinued and replaced with supplemental oxygen via nasal cannula. Cefoperazone–sulbactam sodium was discontinued, and vancomycin monotherapy was continued until discharge. Multiple blood tests showed vancomycin serum concentrations within the normal therapeutic range.

**Figure 4:**
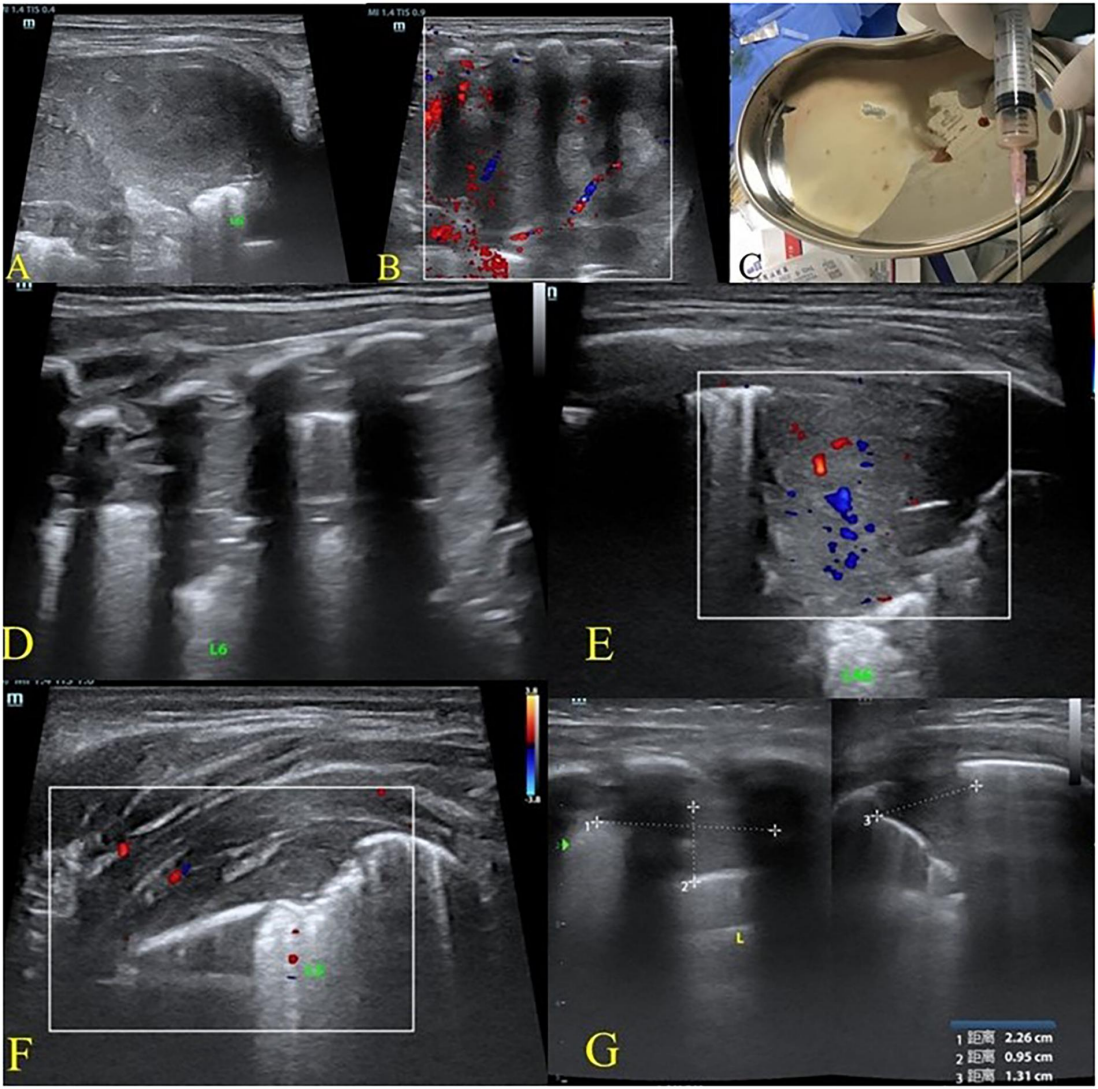
Ultrasound-guided diagnosis and drainage of an enlarged left lower lobe pulmonary abscess on hospital day 14. On hospital day 14, without ventilatory support, ultrasound showed persistent left lung consolidation with a markedly enlarged abscess **(A,B)**. Under ultrasound guidance, percutaneous aspiration and closed thoracic drainage of the left lower lobe abscess were performed, and 10 mL of thick gray pus was aspirated **(C)** On hospital day 29, without ventilatory support, ultrasound demonstrated reduced consolidation in the left lung with complete resolution of the abscess **(D,E)**. Mild pleural thickening was observed on the left side **(F)** Followup ultrasound after discharge showed a small residual consolidation in the left lower lobe without evidence of abscess **(G)**.

Despite continued antimicrobial therapy, serial ultrasound demonstrated progressive enlargement of the left lung abscess. On hospital day 14, LUS (Figures 4A,B) showed no reduction in left lung consolidation and a significant increase in abscess (42 mm × 27 mm × 39 mm), while the right lung improved and consolidation disappeared. On hospital day 15, ultrasound-guided aspiration and closed thoracic drainage were performed, 10 mL of gray white thick liquid was extracted (Figure 4C) and a drainage tube was left. Staphylococcus aureus was detected in the pus. On hospital day16, LUS showed no reduction in left lung consolidation, decreased abscess (23 mm × 17 mm × 16 mm), and a small amount of pneumothorax and viscous fluid accumulation in the left chest cavity.

**Figure 5:**
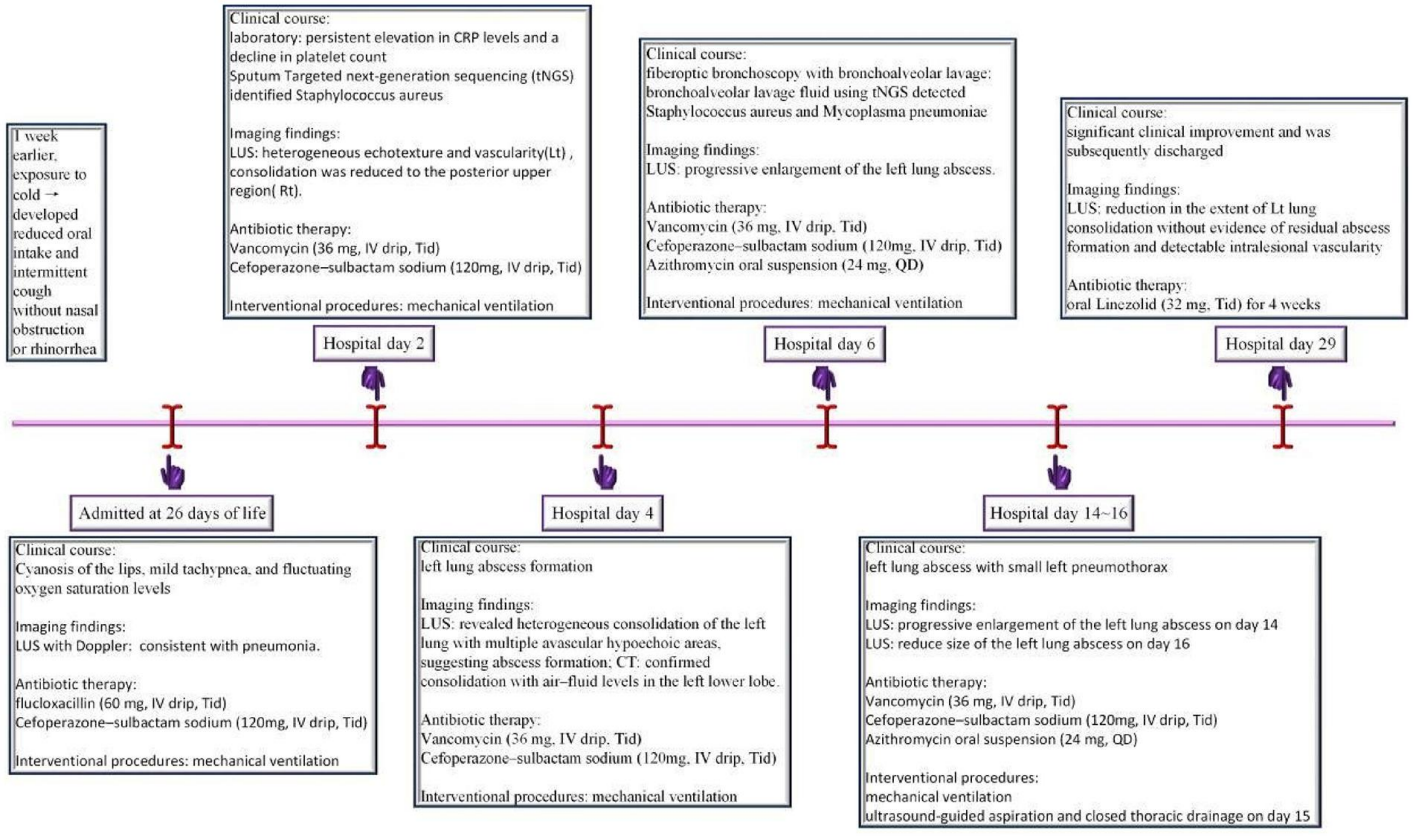
Graphical timeline of the diagnostic and therapeutic workflow during hospitalization, illustrating the clinical course, imaging findings, antibiotic therapy, and interventional procedures.

We continued vancomycin therapy for antimicrobial treatment. By hospital day 29, LUS demonstrated a marked reduction in the size of the consolidation (68 mm × 23 mm × 40 mm), with no evidence of abscess formation and preserved intralesional vascularity (Figures 4D,E). Mild residual pleural thickening was noted (Figure 4F). The infant showed significant clinical improvement and was subsequently discharged. The diagnostic and therapeutic workflow during hospitalization is illustrated in Figure 5. After discharge, the patient received oral linezolid (10 mg, Tid) for 4 weeks for antimicrobial prophylaxis, follow-up ultrasound on postnatal day 88 showed a small residual consolidation (23 mm × 10 mm × 13 mm) in the left lung (Figure 4G). At 6 months of age, CT scan from an external hospital revealed no significant abnormalities in the bilateral lungs. At 15 months of age, the child remained asymptomatic, with normal pulmonary function and growth.

In this study, examinations were conducted with a Mindray M9 portable ultrasound system (Shenzhen Mindray Bio-Medical Electronics Co., Ltd., Shenzhen, China) using a 4–12 MHz linear-array transducer (L12-4s). The infant was examined in the supine, left lateral decubitus, or prone position, depending on the region of interest. A systematic scanning protocol assessed anterior, lateral, and posterior lung regions, subdivided into upper and lower zones. Longitudinal and transverse scans were performed with the transducer perpendicular to the pleura, progressing from sub clavicular to diaphragmatic regions, ensuring comprehensive and reproducible evaluation (5).

## Discussion

This case underscores the expanding and underrecognized role of LUS as a comprehensive diagnostic and management tool in NLA, extending beyond its established application in NP to encompass early detection, dynamic monitoring, and interventional guidance for complex pulmonary infections. While NLA remains a rare entity, its clinical significance is disproportionate due to the potential for rapid deterioration, prolonged hospitalization, and invasive interventions in a highly vulnerable population. Our findings contribute to the growing evidence that ultrasound can fundamentally alter the diagnostic and therapeutic paradigm for NP infections. NLA represents an infrequent but clinically consequential complication of severe NP, characterized by parenchymal necrosis and cavitary abscess formation. Traditional imaging modalities—such as chest radiography and CT—have been mainstays for diagnosis and characterization of lung abscesses. However, reliance on such techniques in neonates is limited by ionizing radiation exposure, the need for patient transport, and logistical challenges, particularly for serial monitoring in critically ill infants. Recent advances in LUS and POCUS are reshaping diagnostic pathways, emphasizing real-time, bedside, radiation-free evaluation and management of pulmonary pathology in neonates and children (3, 12).

### Evolving role of LUS in NP infection

Over the last decade, LUS has transitioned from a complementary tool to a central imaging modality in neonatal respiratory care. Large narrative reviews and multicenter studies demonstrate that LUS has high sensitivity and specificity for NP and its severity, accurately identifying patterns such as irregular pleural lines, B-lines, and subpleural consolidations that predict clinical course and severity (7, 12). The learning of sonographic artifacts—including A-lines versus B-lines, pleural sliding, and air bronchograms—is now fundamental to neonatal respiratory imaging (12).

Importantly, emerging evidence suggests that LUS not only identifies pneumonia but is capable of detecting complicated pulmonary outcomes, such as lung abscess and necrotizing lesions, with diagnostic features that often correlate strongly with cross-sectional imaging. In pediatric cohorts, ultrasound has demonstrated comparable lesion size measurement to CT, reinforcing its utility in both diagnosis and quantitative monitoring (13). Although most large pediatric studies exclude neonates, analogous principles apply given the similar sonographic signatures of cavitary disease.

### Early recognition and dynamic monitoring of NLA

LUS has emerged as a highly sensitive and specific bedside imaging modality for neonatal and pediatric pulmonary infections, frequently surpassing chest radiography in the diagnosis of pneumonia and its complications. Meta-analyses report pooled sensitivities of 96–98% and specificities of 90–94% for LUS in childhood pneumonia, underscoring its diagnostic reliability across diverse clinical settings (14, 15). By enabling dynamic visualization of heterogeneous consolidations, air bronchograms, pleural abnormalities, and early cavitary changes, LUS facilitates real-time detection of disease progression, including the transition from uncomplicated pneumonia to lung abscess, often earlier than conventional radiographs (16).

The strength of LUS lies in its ability to provide serial, real-time assessments of evolving pathology. Bedside scans allow clinicians to distinguish abscess formation from simple consolidation through identification of irregular hypoechoic or anechoic zones lacking internal vascularity (16). Case-based evidence, including point-of-care ultrasound reports in neonates and children, demonstrates that peripheral abscesses can be detected even when initial radiographs are inconclusive, supporting earlier diagnosis and timely management (17).

Longitudinal LUS monitoring provides objective metrics of abscess size and internal architecture, guiding precision-based decisions and interventional therapies while minimizing procedural risk and avoiding cumulative radiation exposure.

### Sonographic features distinguishing La and atelectasis

LUS has become an increasingly valuable bedside imaging modality for evaluating pulmonary pathology, including the differentiation of LA from simple atelectasis, by exploiting distinct sonographic features of consolidated lung tissue. LA are typically identified on LUS as unclear boundary, rounded or oval hypoechoic lesions within areas of lung consolidation, often with internal debris or fluid components and posterior enhancement, consistent with a cavitary infectious process rather than simple collapse; color Doppler may demonstrate peripheral vascular signals around the cavity, further supporting an infectious etiology rather than atelectatic (18, 19). In contrast, atelectasis generally appears as a homogeneous consolidated region without true cavitation, with sonographic signs such as static air bronchograms arising from trapped air in non-ventilated bronchi and absence of significant internal fluid collections or cavitary structures, reflecting alveolar collapse rather than necrotic infection (20). Moreover, the pediatric and neonatal literature emphasizes that careful interpretation of consolidation morphology and associated artifacts can distinguish cavitary processes such as abscess or necrotizing infection from non-cavitating atelectatic collapse, allowing for real-time, radiation-free differentiation that guides clinical decision-making without the need for immediate CT imaging (6).

### Radiation-free serial monitoring and reduction of Ct dependence

Frequent bedside LUS enables continuous, real-time monitoring of evolving pulmonary pathology in neonates without the risks associated with ionizing radiation, a consideration of particular importance in preterm and critically ill infants. LUS permits longitudinal evaluation of abscess dimensions, internal architecture, and adjacent lung recovery, providing objective metrics to guide timely, evidence-based modifications to antimicrobial therapy and supportive care. The modality's non-invasive nature and portability also substantially reduce dependence on CT imaging. Although consensus guidelines increasingly recognize LUS as a first-line tool for neonatal respiratory distress, formal recommendations for complicated infections, such as lung abscess, are still evolving. LUS represents a safe, effective, and dynamic alternative for both diagnosis and longitudinal management in high-risk neonatal populations (21).

### Decision support and ultrasound-guided intervention in NLA

Serial LUS provides objective documentation of persistent or enlarging abscess cavities, enabling precision-based decisions for escalation to interventional drainage rather than relying solely on clinical deterioration. Sonographic features, such as expanding hypoechoic or anechoic regions lacking doppler flow, can justify timely intervention and minimize procedural delays.

Beyond diagnosis and monitoring, LUS facilitates safe and effective ultrasound-guided aspiration and drainage, offering a viable alternative to fluoroscopy- or CT-guided procedures. This is particularly beneficial in neonates, in whom minimizing radiation exposure and maintaining continuous physiological monitoring are of paramount importance. Reports of successful ultrasound-guided abscess drainage with microbiological confirmation underscore LUS's dual diagnostic and therapeutic utility (1).

Ultrasound guidance allows real-time visualization of needle placement, improving procedural accuracy, safety, and diagnostic yield. Pediatric studies demonstrate strong concordance between LUS and CT measurements of abscess dimensions, supporting its use for procedural planning, intervention, and post-procedural follow-up (13). These findings position LUS as an indispensable tool for precision-guided intervention and longitudinal management of neonatal and pediatric pulmonary infections.

### Clinical implications, guideline integration, and future directions

Accumulating evidence supports the expanding role of LUS in NP care; however, current consensus guidelines predominantly focus on pneumonia and pleural diseases, with limited recognition of its utility in complicated infections such as neonatal lung abscess. Clinical data increasingly demonstrate that LUS functions not only as a sensitive diagnostic tool but also as a dynamic modality for monitoring disease progression, guiding interventions, and informing therapeutic decisions. Integration of LUS into standardized clinical pathways could reduce reliance on ionizing radiation, facilitate earlier detection of abscess formation, and support timely escalation of therapy, ultimately improving outcomes for high-risk neonates (22). Bedside, real-time assessment enhances diagnostic efficiency, informs antibiotic stewardship, and enables continuous evaluation of pulmonary status, highlighting LUS as a decision-support tool in neonatal intensive care units (NICUs) and pediatric critical care settings (23).

Future research should aim to establish standardized sonographic criteria for neonatal lung abscess, including thresholds for cavitation size, vascular flow characteristics, and serial changes. Multicenter, prospective studies comparing LUS with CT and clinical outcomes are needed to provide robust evidence for guideline adoption. Collectively, these findings support expanding clinical guidelines to formally incorporate LUS for complicated pulmonary infections, encompassing diagnosis, longitudinal monitoring, procedural guidance, and dynamic decision support, thereby promoting radiation-sparing, patient-centered care.

### Limitations

Several limitations should be acknowledged in this report. First, as a single-center, single-case study, the findings may have limited generalizability to the broader neonatal population, particularly given the unique combination of preterm birth, aspiration, and dual bacterial infection. Second, although lung ultrasound (LUS) provides real-time, bedside evaluation of pulmonary pathology, it is inherently operator-dependent, which may affect reproducibility and interpretation, especially in differentiating heterogeneous consolidations from early cavitary lesions. Third, LUS is primarily sensitive to lesions that abut or are close to the pleural surface; therefore, deep intrapulmonary abscesses without pleural involvement may not be reliably detected, and complementary imaging modalities, such as CT, may still be required in selected cases. Fourth, the absence of standardized quantitative criteria for assessing abscess size and consolidation limits objective comparison across serial examinations. Fifth, while targeted next-generation sequencing (tNGS) enabled pathogen identification, the observed correlation between microbiological results and imaging evolution remains observational and cannot establish causality. Finally, long-term follow-up was limited to discrete clinical and imaging assessments, and subtle pulmonary or functional sequelae beyond 15 months of age cannot be excluded. Collectively, these limitations highlight the need for prospective, multi-center studies with standardized imaging protocols and comprehensive follow-up to further validate the utility of LUS in diagnosing and managing neonatal lung abscesses.

## Conclusion

In summary, the expanding body of evidence positions lung ultrasound as a transformative imaging modality in neonatal lung abscess, transcending its traditional role in pneumonia diagnosis. Through early detection, dynamic monitoring, and procedural guidance, LUS offers a radiation-free, bedside, and patient-centric approach to managing complex pulmonary infections in neonates. Recognition of this expanded diagnostic and therapeutic capacity is essential for advancing evidence-based clinical protocols and improving neonatal respiratory care.

## Data Availability

The original contributions presented in the study are included in the article/Supplementary Material, further inquiries can be directed to the corresponding author.
